# Implications of the COVID-19 pandemic on market orientation in retail banking

**DOI:** 10.1057/s41264-021-00099-9

**Published:** 2021-05-31

**Authors:** Hannele Haapio, Joel Mero, Heikki Karjaluoto, Aijaz A. Shaikh

**Affiliations:** University of Jyväskylä School of Business and Economics, PO Box 35, 40014 Jyväskylä, Finland

**Keywords:** COVID-19, Retail banking, Market orientation, Digital banking

## Abstract

This qualitative study examines the implications of the COVID-19 pandemic on the implementation of market orientation (MO) in the context of retail banking. The findings show that MO was significantly reflected in the behaviors of banks upon encountering the COVID-19 situation, with the banks increasing their MO in response to the crisis. This study finds subcategories based on the empirical data that explain the implementation of MO in more detail. Overall, the findings provide valuable conceptual and managerial insights into the modus operandi of banks during a crisis and offer new best practices for the banking industry.

## Introduction

Market orientation (MO) is a marketing philosophy that perceives marketing as a panorganizational effort to generate market intelligence, disseminate it across the organization, and respond to it in ways that create value for target customers (Kohli and Jaworski 1990). In the context of retail banking, MO necessitates the continuous adaptation of banking services to match customers’ evolving banking needs to create optimal value (Komulainen and Makkonen 2018). While the importance of restructuring organizational operations to match the changing market landscape is self-explanatory, the implementation of MO causes challenges for banks. Many banks claim to be market oriented or customer oriented in their mission statements, yet multiple studies show that bank managers focus more on internal business processes than on external market movement (Camarero 2007; Holmlund et al. 2017; Nätti and Lähteenmäki 2016).

An external crisis offers an opportune time to investigate the implementation of MO because a crisis, by definition, changes the external context of the organization, forcing it to focus on its most critical activities. Accordingly, the COVID-19 pandemic has caused significant changes in retail banking in terms of changing customer needs and internal processes. In particular, COVID-19 has forced retail banks to adopt new digital technologies and boost the use of digital channels for interacting with employees, customers, and other stakeholders. Consequently, the question of how retail banks can remain market oriented in the midst of an unexpected crisis has become vital.

Against this backdrop, this study aimed to explore the implications of COVID-19 regarding the use of MO in retail banking. We followed the seminal MO framework by Kohli and Jaworski (1990) and aimed at generating in-depth insights into the key antecedents, activities, and consequences of MO. Thus, the following three research questions were proposed:

1. Which aspects of MO antecedents have been emphasized in retail banking during the COVID-19 crisis?
2. Which aspects of MO activities have been emphasized in retail banking during the COVID-19 crisis?
3. What are the consequences of COVID-19 for retail banking’s performance?

To reach the study objective, we conducted a qualitative inquiry consisting of ten interviews with managers and advisors from four largest retail banks operating in Finland. Finnish retail banks can be considered forerunners in their degree of digital transformation (e.g., Bank of Finland 2018) because they offer digitized services in all retail banking domains, including transactions, payments, investments, signatures, and service encounters. Consequently, we assumed that Finnish banks were exceptionally well prepared for the COVID-19 crisis in terms of digital maturity, allowing them to focus on business activities rather than adopting new technologies. The study context thus provided a meaningful setting in which to investigate the implications of COVID-19 for MO and potential best practices for addressing such a crisis.

## The impacts of COVID-19 on market orientation

To differentiate themselves from competitors, firms can use MO’s customer-centric business philosophy, which focuses on understanding customer needs and responding to them in ways that create value (Celuch et al. 2015; Jaworski and Kohli 2017). The need to become market oriented has been amplified in the increasingly digitalized, globally competitive, and rapidly changing business environment (Guo et al. 2019; Kohli 2017). In particular, MO has been found to help firms navigate turbulent times (Kumar et al. 2011); therefore, we suggest that MO has provided an important competitive advantage during the COVID-19 pandemic, which has disrupted existing market dynamics.

In addition to adapting retail banking to reflect changes in the market environment, research has demonstrated that a high level of MO in the banking context is positively related to employees’ job attitudes (Gounaris 2003) and customer service quality (Camarero 2007). Thus, although the basic premise of MO is to serve customers via improved offerings based on customer needs, it also improves the commitment of service personnel to providing high-quality customer experiences in service encounters (Edo et al. 2015). Despite the reported advantages of MO and persistent calls for banks to move from a traditional “inward focus” to more market-oriented ways of doing business (see e.g., Kolar 2006), MO implementation has progressed slowly in retail banks. Banks have gradually shifted their attention from firm-oriented risk management to MO (Nätti and Lähteenmäki 2016), yet research shows that bank managers remain largely focused on internal business development and streamlining existing operations (Camarero 2007; Holmlund et al. 2017; Nätti and Lähteenmäki 2016).

Research has also discussed the prerequisites of implementing MO in the banking context. Opoku and Essien (2011) stated that MO requires a bank to involve both managers and employees in implementing an organization-wide focus on customers. Haapio et al. (2019) studied the antecedents of MO in the context of a bank that was undergoing a digital transformation and concluded that the key success factors in leveraging MO stemmed from the manager’s mental model, which genuinely focused on customer needs, and the ability to improve interdepartmental dynamics by removing organizational silos and power games between functions. However, research has provided few insights into the implications of a crisis situation regarding the implementation of MO. Therefore, COVID-19 offered a fruitful opportunity to fill this research gap.

**Fig. 1:**
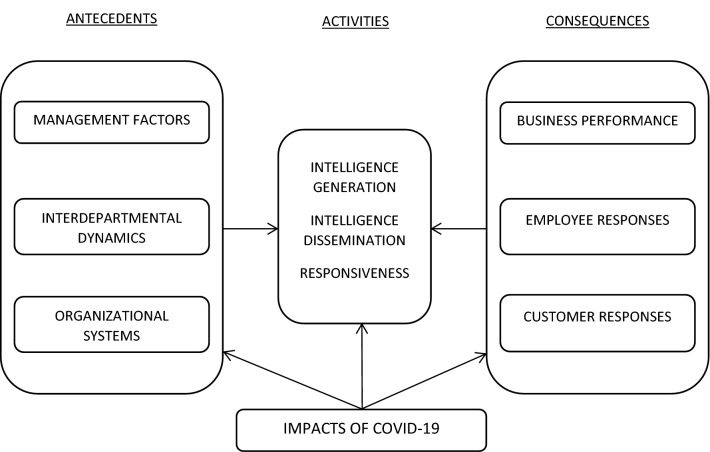
Research framework ( Adapted from Kohli and Jaworski 1990)

Following Kohli and Jaworski (1990), our research framework (Fig. 1) comprises the key antecedents, activities, and consequences of MO. The antecedents are factors that either enhance or impede the implementation of MO. These factors are divided into management factors, interdepartmental dynamics, and organizational systems. MO activities comprise three categories: (1) intelligence generation (i.e., the formal and informal collection of information on market needs and the forces that influence them); (2) intelligence dissemination (i.e., sharing market information within the organization, both vertically across organizational hierarchies and laterally across different functions); and (3) responsiveness to market intelligence (i.e., reconfiguring organizational processes to match changing customer and market needs). The consequences of MO are divided into business performance and employee and customer responses.

The research framework adapted from Kohli and Jaworski (1990) provided a starting point for investigating the implications of COVID-19 regarding the use of MO in retail banking. Three propositions guided our empirical investigation. First, we proposed that increased remote work due to COVID-19 would strengthen the relationship between MO antecedents and MO activities because it becomes increasingly difficult to be market oriented when working remotely without effective managerial practices, close interdepartmental collaboration, and feasible organizational systems. This proposition resonates with studies showing that increased remote work affects interdepartmental dynamics, creates new challenges for management, and challenges existing organizational systems (Bartsch et al. 2020; Carnevale and Hatak 2020).

Second, because the pandemic enforced quick changes to firms’ operations and activities (Finsterwalder and Kuppelwieser 2020; Kabadayi et al. 2020), we proposed that COVID-19 would alter the means through which banks generate market intelligence, disseminate it throughout their organization, and respond to it. Specifically, we suggested that MO activities seen during COVID-19 would lean toward informal information collection and sharing practices to make sense of the rapidly changing environment and quicken responses to market needs.

Third, we proposed that the consequences of COVID-19 on business performance and employee and customer responses would include both positive and negative aspects. In particular, we expected that greater use of digital tools due to COVID-19 would increase operational efficiency but decrease the effectiveness of customer encounters. This proposition aligns with Huhtala et al. (2014), who found that during a downturn, the performance impact of MO increases, but the role of customer orientation decreases.

## Methodology

This study used qualitative, semi-structured, in-depth interviews to examine the implications of COVID-19 for MO. In planning our sample, we sought diversity among the respondents to achieve a holistic picture of the phenomenon. Purposeful sampling (Patton 2002) was used to select the study participants based on three criteria: working at the largest retail banks in Finland; working in different managerial positions and hierarchical levels; and interacting or dealing with retail consumers and/or business customers. In total, six managers and four advisors from different hierarchical levels who work with corporate and/or private customers were selected and interviewed. No new information appeared in the data after the ten interviews; thus, data saturation was achieved (Namey et al. 2016).

The interview guide comprised topics related to our study framework (Fig. 1), following MO’s antecedents, activities, and consequences. Each section started with a general question, followed by more focused questions to extract details. Open-ended questions allowed the interviewees to raise any issues they considered relevant to the topic. Notably, the interviewees were not aware of our study’s theoretical aim, which increased the internal validity of their responses. The interviews were conducted in November and December 2020, lasted 40–56 min each, and were audio-recorded and transcribed verbatim before being coded and analyzed with NVivo. Data analysis followed a two-step thematization process: (1) open coding and (2) evaluation of fit with the theoretical framework. First, we analyzed the verbatim transcripts and created codes to highlight the key issues mentioned by each interviewee. These open codes formed the basis of the coding scheme, which was assessed by multiple authors to ensure internal consistency. This process led to some refinements of the labels and definitions. Second, the theoretical codes identified from the study framework created the basic framework for our empirical findings, where the relevant codes from step 1 were grouped under each of the themes with quotations based on each individual (Table 1). Consequently, our final empirical model presents the key antecedents, activities, and consequences of MO during the COVID-19 pandemic (Table 1).

**Table 1:** Market orientation’s main factors, sub-categories, their descriptions, and example quotes

Main MO factors (based on theory)	Sub-categories for MO factors (based on study data)	Description of sub-categories	Number of respondents	Example quote
**Antecedents**			**10**	
Management factors	1) Empathetic leadership	Caring for employees’ well-being	7	“My first priority is to ensure that all my team members are doing well. It [also] affects customers. When [employees’] well-being is taken care of, they have [the] strength to support customers” (Manager E).
				“Management is now more human. This time period is more human. Managers ask a lot about how I am doing and if everything [is] ok. They do not just stare at results but also pay attention to more human issues” (Advisor A). “The role of leadership has become more important during COVID-19, but first and foremost, the way [that] the managers had prepared their teams to encounter a crisis situation has been the key issue. If you were leading a team that was not self-guided but needed instructions every morning on what they should do, the corona situation would have been a complete disaster” (Manager F).
	2) Employee training	Training employees to take initiative	5	
Interdepartmental dynamic	Internal cooperation	Attitudes and willingness to work as a team with other departments and units and management groups	6	“We were all together in front of a strange threat, and there was no one to blame. Those situations are always fruitful because then you somehow forget everything that is not necessary and focus on things that can drive [you] forward. We forgot the barriers and had the same goal. We were able to show what we are capable of doing together” (Manager E).
Organizational systems	Information technology infrastructure	The constant presence of a reliable and strong digital infrastructure	10	“There are some technical issues that do not completely support the remote way of working. However, we now have quick solutions for these, such as recording all meetings” (Advisor A)
**Activities**			**10**	
Intelligence generation	1) Assessment of customer needs and environmental evaluation	Contacting customers and regulators to discuss their needs in the situation; following competitors	8	“My first reaction was to figure out how we could help our customers through this. I contacted the most important authorities and partners and arranged [for] our people [to] start actively contacting customers” (Manager A).
Intelligence dissemination	1) Formal information sharing	Departmental level	4	“The extended information about [the] situation was created on [the] bank level and then distributed to the organization” (Manager F).
	2) Frequent internal meetings	Departmental/team level	5	“We have [a] meeting every morning with our team. That is important for me” (Advisor D).
	3) Informal communication with coworkers	Individual level	9	“You need [to] be pretty creative not to lose contact with your coworkers. We, for instance, keep the Teams (software) open and have lunch together” (Advisor C).
Responsiveness	1) Fast decision making	Approach: more collaborative and less bureaucratic	5	“Before, we would have nit-picking of tiny details and internal processes. Now, we simply have to act. Normally, for example, creating the customer service sheet on our webpages could have taken a year, and now we made it in few days” (Manager B).
	2) Creativeness	Using innovative thinking and novel methods	3	“The pandemic has forced us to create webinars and [their] content. We have found new tools and methods to [inform] our customers” (Manager D).
**Consequences**			**10**	
Business performance	Negative: Reduced sales revenue	Customers’ unwillingness to make bigger deals (e.g., invest larger amounts of money)	4	“It has affected the customers’ willingness to make decisions. For us, it means that it is harder to get bigger deals when there are fewer face-to-face meetings” (Advisor B)
	Positive: Increased operational efficiency	More customer encounters	6	“The job is now much more efficient. I have more time to meet customers. I have at least doubled the amount of customer meetings when doing those remotely” (Advisor A).
Employee response	Negative: Decreased social connectedness with coworkers	Lack of interactions with other employees and ad hoc discussions	9	“It is a really big disadvantage that you cannot meet [with] your team members [in person] and exchange ideas” (Advisor C)
	Positive: 1) Increased sense of purpose	Work connected to society and customers’ lives	4	“It feels [like] we now do a purposeful job because we see how our work really helps customers and also [has a] larger connection to society” (Advisor B)
	Positive: 2) Increased learning	Forced to change behavior and learn quickly	8	“We were able to show what we are capable [of]. All barriers were broken down, and we had a common goal. Together, as a team, we were thinking [of] how to handle this situation” (Manager E).
Customer response Negative	Negative: Lack of confidence in remote service encounters	Not seeing facial expressions caused uncertainty	4	“Well, certain kinds of personal contact with customers [are] lacking, even though you know the customer well” (Advisor A).
	Positive: Increased convenience	Electronic services are always available and easy to use	6	“Customers adapted to changes very well. Now, we get negative feedback if they need to come and meet us physically, which they claim is difficult” (Manager C).

## Study findings

Overall, the findings of this study confirmed the relevance of the research framework (Fig. 1) in the context of retail banking, implying that the MO theory covered the key behaviors performed by retail banks when facing the COVID-19 crisis. Specifically, we found that the antecedents (i.e., management factors, interdepartmental dynamics, and organizational systems), activities (i.e., intelligence generation, dissemination, and responsiveness), and consequences (i.e., business performance and employee and customer responses) of MO played important roles in retail banks’ efforts to manage the COVID-19 situation. Furthermore, our qualitative data allowed us to divide the main MO antecedents, activities, and consequences into more granular sub-categories, which provided deeper insights regarding the key factors that were perceived as critically important when facing COVID-19. The sub-categories, their descriptions, and example quotes can be found in Table 1.

### Antecedents of MO

In terms of *management factors,* the interviewees perceived that COVID-19 had significantly increased the importance of empathetic leadership and employee training. It was clear that managers had become more empathetic toward their employees and understood that they were experiencing a new and difficult situation. Therefore, the managers focused on ensuring their employees’ well-being, which was considered to ultimately be reflected in service encounters. The managerial focus on employees’ well-being was heavily emphasized by the managers but also supported by multiple advisors who appreciated the change in their managers’ attitudes during COVID-19.

The role of employee training also gained importance during COVID-19. As remote work increased, the employees could not be supervised and guided in real time. Thus, employees had to possess the necessary competencies and knowledge to take initiative and make independent decisions. Notably, training employees to take initiative is a long process, making it difficult to react to a crisis situation via training. Regular training of employees is needed to be prepared for a crisis.

The effects of COVID-19 on *interdepartmental dynamics* were perceived differently between advisors and managers. The advisors reported that the focus on avoiding silos has been an ongoing issue and has nothing to do with COVID-19, while the managers found it a remarkable improvement concerning cooperation between different organizational departments and units. Furthermore, all the respondents considered effective *organizational systems* a vital prerequisite for implementing MO during COVID-19. Specifically, the information technology infrastructure supported the shift to remote work, and its absence would have led to serious problems in service delivery and other operational tasks. However, it was also mentioned that the situation revealed minor technical shortcomings.

### MO activities

The key activities based on the framework were highlighted by all respondents with the following emphasis on COVID-19. Regarding *intelligence generation*, banks were quite proactive in making sense of the crisis (i.e., they tried to respond to an unexpected development by noticing and bracketing it, establishing a shared understanding of it, and attempting to create a more ordered environment to draw further cues). To do this, banks proactively contacted customers, partners, regulators, and other stakeholders as well as followed what their competitors were doing.

COVID-19 also affected *intelligence dissemination* by increasing the frequency of internal meetings at different levels of the organization. In particular, the managers reported having significantly more frequent meetings with their supervisors and colleagues to share insights on market events and the concerns of customers and other stakeholders. Frequent meetings at the senior management level enabled managers to disseminate information further to their employees and prepare them for customer encounters. However, some advisors reported that the lack of informal meetings posed a challenge, stating that they missed informal knowledge sharing with coworkers, which highlights the critical role of information dissemination during COVID-19.

Our findings revealed that COVID-19 has had significant implications for *responsiveness to market intelligence*. All the interviewed banks had attempted to accelerate their decision-making processes, particularly in terms of responding to customer concerns. This move was supported by swift reconfiguration of internal processes and adaptations to new practices. In particular, employees and customers adapted to the use of remote channels quickly. Every interviewee reported that the move to “remote mode” was rapid, both internally and externally. Furthermore, some respondents mentioned that they started to use innovative thinking to quickly create new ways to respond to the situation (e.g., using interactive webinars instead of customer events or forming small customer groups to test and discuss needed services virtually).

### Consequences of MO

Overall, the increased MO as a result of COVID-19 led to positive business consequences. However, the crisis also induced negative effects that could not be overcome with increased MO. In the following, we discuss the consequences for business performance, employee responses, and customer responses.

The most commonly mentioned positive effect on *business performance* was the increased efficiency of service delivery. Most managers and advisors agreed that they now have more customer encounters than before because the remote meetings are more efficient compared to physical meetings. Two of the banks’ respondents claimed that they had attracted many new customers during the crisis. One negative aspect noted was that many customers were not ready to make decisions concerning larger investments remotely. Instead, these customers wanted to wait until the pandemic waned to allow face-to-face discussion (offline). This might be partially (yet not totally) explained by a lack of trust; this challenge was even reported for customers who already had a long-term relationship with their advisor. Such a challenge will naturally affect a bank’s business results.

The findings related to *employee responses* were largely positive. Specifically, most respondents felt more motivated toward their work and realized its importance to society.

Another positive employee response was learning and self-improvement. For managers, learning how connectedness and commitment helped them overcome challenges was especially significant. Negative comments concerned the lack of social contact. While some employees had done remote work before, it was a novel way of working for others. However, most interviewees reported that they missed social contact with colleagues, especially ad hoc discussions, making them feel quite lonely at times.

In terms of *customer responses*, most interviewees reported that customers had given positive feedback, especially regarding the convenience of using electronic services and general satisfaction. Reasons for such positive feedback included customers’ willingness to use electronic services, bank employees’ attitudes, and the increased number of contacts. The negative customer responses were related to the lack of facial expression in remote meetings. Video meetings partly solved this problem, but not all customers wanted to use video. This may be partly explained by all the advisors stating that they also found it harder to build trust with customers in remote meetings due to the inability to see faces and gestures.

## Discussion and conclusion

The present study was designed to determine the effects of COVID-19 on the implementation of MO antecedents, activities, and consequences in retail banking. This study contributes to marketing theory from the MO perspective by discussing the implications of COVID-19 in the retail banking industry. We found that during a crisis, such as COVID-19, MO is a relevant approach. As mentioned in the literature review, banks are shifting from risk management to focusing on customers (Nätti and Lähteenmäki 2016), which this study broadly supports and is evident in the highlighted antecedents and activities. Empathetic leadership, employee training, excellent cooperation between and among different departments and teams, and technological readiness and capability are seen as necessities for creating value for customers.

MO activities, such as understanding the situation by increasing interactions and discussions with customers and partners and by reviewing the strategies of competitors and regulators, clearly focus on creating customer value.

Hence, our results emphasize the importance of both formal and informal communication and the need for social connectedness to focus on customers’ needs and wants. Another interesting finding was that the activities show connections to entrepreneurial orientation (EO), especially regarding the promptness, flexibility, and use of ad hoc information. This finding is consistent with that of Morgan and Anokhin (2020), who claimed that large firms, especially when experiencing environmental turbulence, could benefit from implementing EO and MO simultaneously.

Our study shows both negative and positive consequences of implementing MO, as proposed. The main finding regarding the former was that the connection to business performance is unclear. While the volume of customer meetings increased, there were negative comments in terms of the results of those remote meetings. This finding aligns with the results of several earlier studies (e.g., Gerrard et al. 2006; Levy and Hino 2016), proving that customers value human interaction. Regarding the latter, the consequences for employees and customers were mainly positive. Employees felt that their work became more meaningful, and managers were more caring and willing to learn about employees’ work and situation than before. Hence, customers were satisfied when banks proactively contacted them to learn about their situation and needs and advised them on using remote services. This involvement enabled customers to quickly receive new and relevant services that were developed to help them during COVID-19

In general, our study shows that COVID-19 has become a major driver of digital transformation in retail banks. This aligns with Baicu et al. (2020), who found a strong increase in the consumption of mobile and Internet banking during the pandemic. As mentioned in introduction, Finnish banks can be seen as forerunners in digitalization and adjusting delivery channels, which prepared employees and customers in the Finnish banking sector for the change and helped them easily adapt to remote services. Our results align with those of Tiirinki et al. (2020), who stated that the consequences of the pandemic will be drastic in Finland yet perhaps less dramatic and extensive than in other Western industrial countries.

Taken together, these results suggest that the implementation of MO is useful in expanding our understanding of retail bankers’ behavior during times of crisis as well as acting as a launchpad for business beyond the current pandemic and other crises.

### Managerial implications

This study provides several implications for practice. For example, banks must stay connected with their customers and other stakeholders. Maintaining regular contact with customers during the new normal is based on simple logic: provide comfort and peace of mind, gain an understanding of what is happening in their lives, and determine what actions would most help them through a crisis. Thus, the capacity building of employees during a crisis should be at the core of this strategy to ensure uninterrupted delivery of services. This study also highlights the importance of managers staying connected to their employees and being absolutely present. Our results show that the pandemic has strongly affected management and employee mindsets. A joint strategy involving employees working in different units and teams could perhaps offset challenges and crises effectively and efficiently.

### Limitations and future research directions

As with any qualitative study that is conducted in a specific context, this study is not without limitations. The study explored the effects of COVID-19 in the context of retail banking in Finland, focusing on the implementation of MO. Thus, the findings highlight views and prerequisites in retail banking only and cannot be generalized to other services in other contexts. However, the participants represent a heterogeneous group of people with diverse experiences in retail banking services, which ensured extensive and versatile data with plausible outcomes related to the studied phenomenon. In addition, the research was conducted, while the pandemic was ongoing. Thus, future studies could assess the long-term effects. Further research is required to determine whether the studied behavior represents a permanent change and, if so, how the consequences evolved.
